# Autologous Peripheral Blood-Derived Orthobiologics for the Management of Hip Osteoarthritis: A Systematic Review of Current Clinical Evidence

**DOI:** 10.7759/cureus.70985

**Published:** 2024-10-07

**Authors:** Ashim Gupta, Anish G Potty

**Affiliations:** 1 Regenerative Medicine, Future Biologics, Lawrenceville, USA; 2 Orthopaedics, South Texas Orthopaedic Research Institute, Laredo, USA

**Keywords:** autologous conditioned serum, autologous peripheral blood-derived orthobiologics, autologous protein solution, gold-induced cytokine, hip, hyperacute serum, osteoarthritis, plasma rich in growth factors, platelet lysate, regenerative medicine

## Abstract

Osteoarthritis (OA) of the hip affects millions of people with a sizable health-related economic burden. Conventional treatment modalities are prioritized, turning to surgical intervention only when they have failed. Nevertheless, these approaches have flaws, regularly trying to provide symptomatic pain relief instead of focusing on the underlying etiology. The last two decades have seen a significant increase in the use of autologous peripheral blood-derived orthobiologics (APBOs) for managing musculoskeletal disorders, including OA of the hip. Platelet-rich plasma (PRP) is the most regularly used APBO. Yet, studies have shown its inefficacy in improving pain and function along with a high incidence of reporting bias in systematic reviews and meta-analyses involving PRP injections for hip OA. Thus, the potential of using other APBOs, including platelet lysate (PL), autologous conditioned serum (ACS), gold-induced cytokine (GOLDIC), plasma rich in growth factors (PRGF), autologous protein solution (APS), and hyperacute serum (HS), for managing OA of the hip was investigated. This review summarizes the results of clinical studies involving the mentioned APBOs to manage OA of the hip. Multiple databases (Scopus, Embase, PubMed, and Web of Science) were searched employing terms for these ‘APBOs’ and ‘OA of the hip’ for articles published in the English language till September 21, 2024, adhering to the Preferred Reporting Items for Systematic Reviews and Meta-Analyses (PRISMA) guidelines. Only two articles fit the scope of our study, and both included articles involved the use of ACS. No clinical studies involving the use of PL, GOLDIC, PRGF, APS, and HS were identified. No ongoing clinical trials were listed on any of the searched registers involving the use of the aforesaid APBOs. Intra-articular administration of ACS is safe and can reduce pain in patients with OA of the hip. Nonetheless, given the dearth of pertinent literature and limitations of included articles, more adequately powered, prospective, multicenter, controlled, open-label or blinded, randomized, and non-randomized trials with extended follow-up are necessary to determine the efficacy of various APBOs for managing hip OA. Further comparative studies to assist clinicians in finding the ideal APBO for the treatment of OA of the hip are needed.

## Introduction and background

Osteoarthritis (OA) is the most widespread form of joint disorder, typically affecting large weight-bearing joints such as knees and hips [1-3]. It involves degenerative changes in the joint cartilage, resulting in pain, stiffness, and restricted motion [1-3]. OA is the leading cause of chronic disability and health-related economic burden across the world [1-3]. Conventionally, the inherent cause of OA was assumed to be joint overuse and degenerative changes; however, recent articles have suggested the role of other factors, including genetics, biomechanical variability, pro-inflammatory cytokines, and metabolic factors [4,5]. The current modalities for the treatment of OA of the hip include weight management, exercise, walking aids, non-steroidal anti-inflammatory drugs (NSAIDs), intra-articular administration of steroids and hyaluronic acid, and surgical intervention, when necessary [6,7]. However, these approaches can sometimes overlook the underlying etiology of the symptoms and miss severe diagnostic conditions such as cancer [6-8].

The last two decades have seen a significant increase in the use of autologous peripheral blood-derived orthobiologics (APBOs) for managing musculoskeletal disorders, including OA, attributed to their regenerative and anti-inflammatory properties [9,10]. Platelet-rich plasma (PRP) is the most regularly used APBO; yet, several systematic reviews and meta-analyses reported no superiority of PRP in terms of alleviating pain and increasing function in patients with OA of the hip compared to the control [11-13]. In addition, a recent study also reported a high prevalence of reporting bias in systematic reviews and meta-analyses of PRP injections for hip OA [14]. The study outcomes further suffer due to variations in PRP preparation protocols and associated characterization, and patient variables, such as age, medications, and comorbidities [13-15]. Specifically, the variability in PRP composition (platelet concentration compared to the baseline whole blood levels, absolute platelet count, and presence or absence of leukocytes and red blood cells) leads to differing clinical outcomes [16-18]. To sidestep the limitations posed by PRP, the potential of using other APBOs, including platelet lysate (PL), autologous conditioned serum (ACS), gold-induced cytokine (GOLDIC), plasma rich in growth factors (PRGF), autologous protein solution (APS), and hyperacute serum (HS), for managing OA of the hip was investigated [16,19-23]. Nonetheless, to date, there are no reviews summarizing the findings of clinical studies examining the effectiveness of these APBOs for the management of OA of the hip. Thus, the primary goal of this review is to summarize the results of clinical studies involving the mentioned APBOs for the treatment of hip OA. The secondary goal is to list the ongoing clinical studies registered on different clinical trial protocol repositories involving these APBOs for managing OA of the hip.

## Review

Methods

Search Criteria

**Figure 1:**
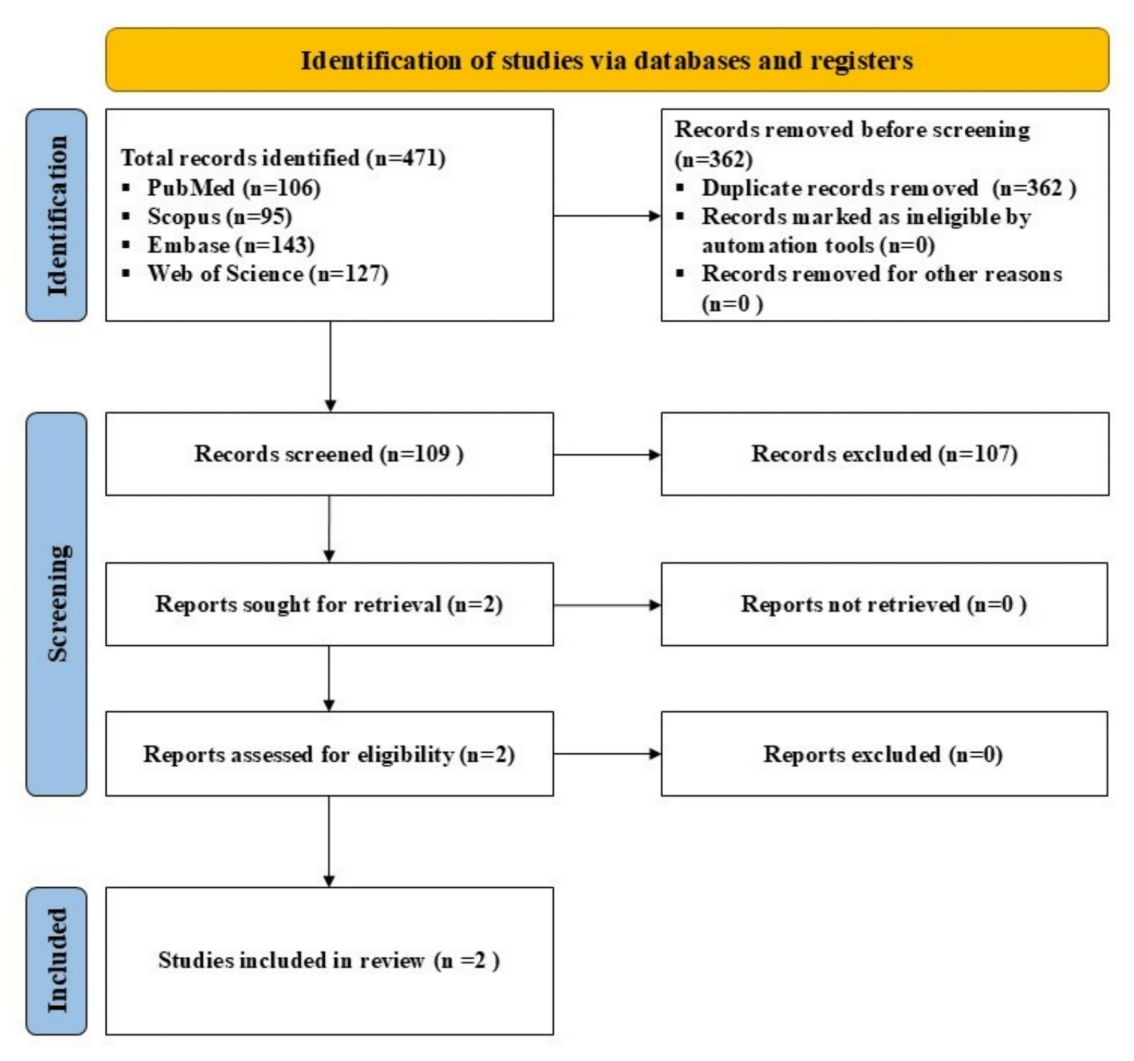
A PRISMA flow diagram outlining the record identification and selection process PRISMA: Preferred Reporting Items for Systematic Reviews and Meta-Analyses

A search was made using the terms, (‘platelet lysate’ OR ‘PL’) or (‘autologous conditioned serum’ OR ‘ACS’) or (‘gold-induced cytokine’ OR ‘GOLDIC) or (‘plasma rich in growth factors’ OR ‘PRGF’) or (‘autologous protein solution’ or ‘APS’) or (‘hyperacute serum’ OR ‘HS’ OR ‘hypACT’) AND (‘hip’) or (‘osteoarthritis’) or (‘hip osteoarthritis’) in multiple databases (Scopus, PubMed, Web of Science and Embase), for studies published till September 21, 2024, in the English language, while adhering to Preferred Reporting Items for Systematic Reviews and Meta-Analyses (PRISMA) guidelines. All clinical studies involving these APBOs to manage OA of the hip were included (Figure 1). Studies not involving the aforesaid APBOs alone or not targeting OA of the hip were excluded. The comparators can be baseline, placebo, and/or active modalities. The outcome measures comprised patient-reported outcome measures (PROMs) and/or other clinical measures.

To curtail the risk of bias, all authors discussed and reviewed all the selected studies, references, and excluded articles. Any dissents were resolved after a thorough discussion. All the data were extracted and analyzed by the first author and then reviewed and approved by the last author.

In addition, ongoing clinical studies concerning the utilization of the APBOs for managing OA of the hip, registered on various clinical studies repositories, including ClinicalTrials.gov, Clinical Trials Registry - India (CTRI), and Chinese Clinical Trial Register (ChiCTR) using the search terms, were documented.

Results

Platelet Lysate (PL)

PL is obtained from PRP and prepared via a double freeze/thaw cycle (freeze at -80^o^C and thaw at 37^o^C) [16,19]. Up till now, there are no published clinical studies involving PL for the treatment of hip OA.

Autologous Conditioned Serum (ACS)

ACS is an acellular preparation formulated by incubating whole blood in a syringe (containing medical-grade glass beads) at 37^o ^C for 24 hours, followed by centrifugation of the blood to collect serum [19,20]. Two studies involving ACS for managing OA of the hip met our inclusion criteria (Table 1).

**Table 1:** Summary of main findings of included clinical studies involving autologous conditioned serum for the management of osteoarthritis of the hip ACS: Autologous Conditioned Serum; VAS: Visual Analog Score; IL-1RA: Interleukin-1 Receptor Antagonist

Author (Reference)	Type of Study	Sample Size	Age and Sex	Treatment	Main Findings
Baltzer et al. [24]	Retrospective, clinical, non-blinded, non-randomized intervention study	119 patients (150 hips)	Age: 62.08±0.71 Females: 51.3% Males: 48.7%	Groups: ACS only; ACS + cortisone; and ACS + cortisone + IL-1RA	Intra-articular administration of ACS in patients with OA of the hip is safe and led to significant improvement in pain (VAS scale) at the 14th-month follow-up compared to the baseline, and additional injections of steroids and IL-1RA did not enhance the treatment effect of ACS alone.
Tassara et al. [25]	Retrospective case report	3 hip patients out of 28 cases	Not provided for hip patients	ACS	Intra-articular administration of ACS in patients with OA of the hip is potentially efficacious in terms of reducing pain (VAS scale) at the sixth-month follow-up compared to the baseline.

Baltzer et al., in a retrospective, non-randomized, non-blinded, interventional clinical study, investigated the efficacy of intra-articular injection of ACS on patients with OA of the hip and determined whether the likely treatment outcome can be enhanced by supplementary injections of recombinant interleukin-1 receptor antagonist (IL-1RA) and steroids [24]. The inclusion criteria included patients over 30 years of age, clinical evidence of OA of the hip per the judgment of the clinician, the presence of pain and disability, and radiographic analysis (Kellgren Lawrence hip grade 2-4). The exclusion criteria included the presence of active infection, cancer, abnormal hematological reports, and poor general health. ACS was formulated using a commercial kit (Orthogen, Germany) per the manufacturer’s instructions. A total of 119 patients (150 hips) were allocated to one of the 3 groups - ACS only (62 hips; 5.94 injections of 2 mL ACS), ACS + cortisone (71 hips; 5.7 injections of 2 mL ACS + 1.94 injections of 10 mg Triamcinolone, each), and ACS + cortisone + IL-1RA (17 hips; 5.88 injections of 2 mL ACS + 2.88 injections of 10 mg Triamcinolone + 3.53 injections of 0.2 mg IL-IRA, each). The PROMs included a visual analog scale (VAS) score, assessed at baseline and at about the fourteenth-month follow-up after the last injection. All groups showed statistically significant improvements in the VAS score at the fourteenth-month follow-up compared to the baseline, independent of the severity of the OA. The addition of cortisone or cortisone + IL-1RA did not add any benefit over the treatment with ACS alone. The limitations of this study include the absence of placebo control and blinding, small cohort size, and short follow-up. The administration of ACS is safe and led to significant improvement in pain, and additional injections of cortisone and IL-1RA did not increase the treatment efficacy of ACS alone.

Tassara et al., in a retrospective report, investigated the effectiveness of intra-articular injection of ACS for the treatment of OA [25]. Out of 28 cases included in this study, only 3 cases were of OA of the hip (the rest were for OA of the knee). The inclusion criteria included patients who are ≥18 years old, agreed to the treatment with ACS, completed all 4 injections of ACS, and VAS before treatment was 50 mm or more. The exclusion criteria included patients with documented infected joints, neurological/psychiatric conditions, and vascular and infectious diseases. ACS was formulated using a commercial kit (Orthogen AG, Düsseldorf, Germany) per the manufacturer’s instructions, and 2 mL once a week over four weeks was administered. The outcome measure included VAS, assessed at baseline and at the one- and six-month follow-up. All three patients with OA of the hip showed consistent improvement in pain at follow-up time points compared to the baseline. The limitations of this study include the short follow-up and the very small sample size. The Administration of ACS is potentially efficacious in terms of reducing pain in patients with OA of the hip.

Gold-induced Cytokine (GOLDIC)

GOLDIC is a kind of ACS formulation that involves the incubation of whole blood with gold particles [19]. Till now, there are no published clinical studies involving GOLDIC for the treatment of hip OA.

Plasma Rich in Growth Factors (PRGF)

PRGF is prepared by activating red blood cell-depleted and leukocyte-poor PRP with calcium chloride [19]. Till now, there are no published clinical studies involving PRGF for the treatment of hip OA.

Autologous Protein Solution (APS)

APS is formulated by incubating leukocyte-rich PRP with polyacrylamide beads [19,21]. Till now, there are no published clinical studies involving APS for the treatment of hip OA.

Hyperacute Serum (HS)

HS is prepared by mechanically secreting, via pressing or centrifugation, cytokines and growth factors from the platelet-rich fibrin clot [19,22]. Till now, there are no published clinical studies involving HS for the treatment of hip OA.

Ongoing Clinical Trials

As of September 21, 2024, no clinical trials were listed on ClinicalTrials.gov, CTRI, or ChiCTR to evaluate the safety and/or effectiveness of these APBOs to manage OA of the hip.

Discussion

This review examined the potential efficacy of several APBOs, such as PL, ACS, GOLDIC, PRGF, APS, and HS, for the treatment of OA of the hip. All clinical studies utilizing these APBOs for managing OA of the hip were included. Only two articles, based on our search and inclusion/exclusion criteria, fit the scope of our study. Particularly, both included articles involved the use of ACS. No clinical studies involving the use of PL, GOLDIC, PRGF, APS, and HS were identified. No ongoing clinical trials were listed on the searched registers involving the use of these APBOs.

Cytokines play a vital role in the detrimental cascade in OA with interleukin (IL)-1 being the most potent initiator of degeneration [26]. IL-1 works by downregulating the expression of proteoglycans and upregulating the expression of matrix metalloproteinases [26]. Several efforts have been made to develop the curative use of IL-1 inhibitors, including IL-1RA, type 1 cytokines, such as IL-4, -10, and -13, and soluble forms of IL-1 receptors that can prevent the IL-1 synthesis and promote IL-1RA synthesis [25]. To accomplish this, ACS was developed (first by Orthogen), resulting in at least a 100-fold increase in the levels of IL-1RA along with other anti-inflammatory cytokines [27-30]. However, no significant rise in the level of the pro-inflammatory cytokine, including tumor necrosis factor-α and IL-1β, was observed [31]. It is hypothesized that the clinical benefits of ACS are attributable to the synergistic relationship between IL-1RA and other cytokines though the exact mechanism of action is yet to be fully understood [32]. The effectiveness of ACS was first evaluated for the treatment of knee OA [33], but later, this was extended to other musculoskeletal conditions, including OA of the hip [24,25,34-36]. Baltzer et al. showed that injection of ACS intra-articularly in patients with OA of the hip is safe and resulted in significant improvement in pain at 14 months of follow-up compared to the baseline [24]. Moreover, supplementary administration of steroids and/or IL-1RA did not augment the treatment efficacy of ACS [24]. Tassara et al. showed that intra-articular administration of ACS in patients with OA of the hip is potentially efficacious in reducing pain at the sixth-months follow-up though the very small sample size restricted a more in-depth analysis of the data [25].

This review has pitfalls, including the inclusion of only two clinical studies and the involvement of the use of ACS only, among many APBOs. This lessens the capability to critically evaluate the effectiveness of individual APBOs for managing OA of the hip. In addition, the included studies have deficits, including small cohort size, short follow-up, and absence of placebo and/or active comparators. Moreover, the risk of publication bias due to the higher likelihood of publication of studies with positive outcomes can result in insufficient analysis of their complete effectiveness [37].

## Conclusions

The intra-articular administration of ACS is safe and can reduce pain in patients with OA of the hip. Nonetheless, given the dearth of pertinent literature and limitations of included articles, more adequately powered, prospective, multicenter, controlled, open-label or blinded, randomized, or non-randomized trials with extended follow-up are necessary to determine the efficacy of various APBOs for managing hip OA. Further comparative studies to assist clinicians in finding the ideal APBO for the treatment of OA of the hip are also needed.
